# Supplementary material for: Nanoscale electric-field imaging based on a quantum sensor and its charge-state control under ambient condition
Source: Nat Commun. 2021 Apr 28;12:2457. doi: 10.1038/s41467-021-22709-9 (PMC8080810; doi:10.1038/s41467-021-22709-9)
Supplement: Supplementary file 1 — Supplementary Information [file 41467_2021_22709_MOESM1_ESM.pdf]

Supplementary Information

Nanoscale electric-field imaging based on a quantum sensor and its charge-state control under ambient condition

Ke Bian[ξ], Wentian Zheng[ξ], Xianzhe Zeng, Xiakun Chen, Rainer Stöhr, Andrej Denisenko, Sen Yang, Jörg Wrachtrup[*], Ying Jiang[*]

[ξ]These authors contributed equally to this work.

[*]Corresponding author. E-mail: j.wrachtrup@pi3.uni-stuttgart.de (J.W.); yjiang@pku.edu.cn(Y.J.)

This PDF file includes:

Supplementary Note

Supplementary Figs 1-7

References

Supplementary Note 1: Simulation of the scanning gradient-field images

In our system, we used NV^- associated with N^{15} isotope as probes. Instead of employing nuclear spin state m_I=0 for sensing the external electric field[1,2], we measured the field strength from the frequency shift of $m_I=\pm 1/2$ states (Fig. 1d). The spin Hamiltonian of ground triplet states of NV^- under the magnetic field, electric field and hyperfine interaction can be written as:

$$H_{gs}=H_{el}+H_{hf} \tag{1}$$

where

$$H_{el}=(D+k_{\parallel}E_{\parallel})S_z^2+\gamma_e\vec{\mathbf{B}}\cdot\vec{\mathbf{S}}-k_{\perp}E_x\left(S_x^2-S_y^2\right)+k_{\perp}E_y\left(S_xS_y+S_yS_x\right)$$

$$H_{hf}=A_{\parallel}S_zI_z+A_{\perp}(S_xI_x+S_yI_y) \tag{2}$$

D is the zero-field splitting strength (2870 MHz) of NV^-, γ_e=2.8 MHz Gauss^{-1} is the gyromagnetic ratio of electron, $k_{\parallel}$=0.35±0.02 Hz·cm V^{-1} and $k_{\perp}$=17±3 Hz·cm V^{-1} are the electric-field coupling strength for parallel (axial) and transverse (nonaxial) components, $A_{\parallel}$=3.03(3) MHz and $A_{\perp}$=3.65(3) MHz are the N^{15} hyperfine parameters[3]. The coordination of NV is denoted in Supplementary Fig. 1a. In the presence of transverse magnetic field, the eigenstates of NV^- change into an equal mixture of $m_s=\pm1$ spin projection, i.e. $|\pm\rangle$ states. Considering that the frequency shift caused by external field (<1 MHz) is relatively small compared to the hyperfine coupling strength ($A_{\parallel}$ ~3.03 MHz), we solved the approximate eigenvalues of H_{gs} to the second-order correction:

$$E_0^{(2)}=-2\Lambda$$

$$E_{\pm}^{(2)}=D+k_{\parallel}E_{\parallel}+\Lambda+K\pm\left(k_{\perp}^2E_{\perp}^2-2\Lambda k_{\perp}E_{\perp}\cos\phi+\Lambda^2+K^2+2\Lambda K+\frac{A_{\parallel}^2}{4}\right)^{\frac{1}{2}} \tag{3}$$

Where $B_{\perp}=\left(B_x^2+B_y^2\right)^{1/2}$, $E_{\perp}=\left(E_x^2+E_y^2\right)^{1/2}$, $\Lambda=\gamma_e^2B_{\perp}^2/2D$, $K=(A_{\parallel}^2+4k_{\perp}^2E_{\perp}^2)/8D$, $\phi=2\phi_B+\phi_E$, $\tan\phi_B=B_y/B_x$, and $\tan\phi_E=E_y/E_x$. Since $A_{\perp}\ll D$, the mixing of states with different nuclear projections has very small effects on zero-field hyperfine structure, we neglected the contribution from $A_{\perp}$. Both the strength of transverse

magnetic field applied by the vector magnet and the electric field of tip could be estimated through the spin resonance spectra using Supplementary Eq. (3). Compared with the transverse component $E_{\perp}$, the parallel component $E_{\parallel}$ has negligible effects on the shift of spin resonance due to $k_{\parallel} \ll k_{\perp}$. Therefore, we can ignore the contribution from $E_{\parallel}$. According to Supplementary Eq. (3), the strength of the transverse electric field $E_{\perp}$ sensed by the NV in Fig. 2b was estimated to be 119±3.3 kV cm^{-1}, averaged out of all possible ϕ_{E}. In addition, we calculated $B_{\perp}$=9.6 Gauss using pulsed-ODMR measurements under zero electric-field in Supplementary Fig. 1d.

Next, we simulated the field-gradient imaging with the same experimental parameters as in Fig. 2b. We simply modeled the conducting tip as a triangular pyramid with equal sides and a finite spherical tip apex at the end, which is consistent with FIB cutting process (see inset in Fig. 2a). Then we simulated its electric field distribution by COMSOL (Fig. 2d). The frequency shifts arising from the projected $E_{\perp}$ and $E_{\parallel}$ were calculated through Supplementary Eq. (3) and convolved with the experimental pulsed-ODMR spectra. We fit the pulsed-ODMR resonant peak by the Lorentz function:

$$P=\frac{a}{\left[\frac{2(f-f_0)}{f_{\mathrm{FWHM}}}\right]^2+1} \quad (4)$$

with a=-0.11 and f_{FWHM}=260 kHz, which were extracted from the experimental data acquired by the same NV in Fig. 2b and c. The simulated scanning field-gradient image (Fig. 2e) agrees well with the experimental one (Fig. 2b). However, the bias voltage of the tip used in the simulation (-9.6 V) is somewhat smaller than that in experiment (-16 V). We attributed this to the screening effect resulting from the defect donors, hydration layers or induced ions by high voltage near diamond surface[4], which is not considered in our model. It is possible to quantitatively extract the surface screening effect by modeling the dielectric response of the surface charges on the diamond.

Finally, we simulated the field distribution along the surface normal (E_z, denoted as the gray arrow in Fig. 2a) of the same tip model. We could obtain the maximum field strength of 14 MV cm^{-1} when tip is right above a NV center with the depth of 5 nm (Fig. 2d). The positioning precision of NV center through scanning field-gradient imaging is

~13.9 nm, which is limited by the field gradient of tip and the spectral broadening of pulsed-ODMR (Fig. 2f).

Supplementary Note 2: Estimation of electric noise from a charged oscillating AFM tip

Once the tip or the sample is electrically charged, the noise induced by the oscillating AFM tip can significantly degrade the NV coherence. In order to estimate the effect of tip oscillation on the ODMR line broadening, we adopt the conditions in Fig. 2b, where the tip voltage is -16 V, the horizontal tip-NV distance is about 100 nm, and the field spatial resolution is ~10 nm. Considering the field sensitivity of our NV system (~17.6 kV cm^{-1}), the estimated field gradient is ΔE=1.8 kV cm^{-1} nm^{-1}.

Next, we ignored the contribution from the parallel component of external field $E_{\parallel}$ to the spin energy level shift and assumed roughly that the field gradient is homogeneous. Then, we obtained the variation of frequency shift (Δf) under an A.C. electric field induced by the oscillating tip:

$$\Delta f = 2A\Delta E \cos\theta\, k_{\perp} \quad (5)$$

where A is the amplitude of tip oscillation, $k_{\perp}$=17 Hz cm V^{-1}, and θ is the angle between [100] and [111] direction of the diamond sample with $\cos\theta \approx$0.82. This relation suggests that a tip amplitude of ~5 nm will lead to a spectral broadening of 251 kHz, which is comparable to the intrinsic FWHM of the pulsed-ODMR spectra (~300 kHz in our case). Considering that the oscillation amplitude of conventional NV-AFM is typically well above 5 nm, the induced noise will undoubtedly affect the electric-field sensing based on the NV center.

Supplementary Note 3: Highly efficient control of charge state

In order to achieve highly efficient control on the NV charge state, it is necessary to locate the AFM tip to the proximity of NV with nanoscale precision. Thanks to the weak scattered light from the AFM tip, we can roughly guide the tip close to the selected fluorescent NV spot through the confocal image (Supplementary Fig. 2b). The tip can

be also positioned on the NV by simultaneously recording the AFM force signals (frequency shift) when acquiring the confocal image (Supplementary Fig. 2a). An obvious frequency-shift change will appear at the position of tip because of the laser-induced thermal expansion under high laser powers (typically >200 μW in our experiments). In our case, the scanning speed of laser focus with a diameter of ~300 nm is typically 20~40 μm s^{-1}, which is faster than the PID parameter of AFM feedback in constant-frequency shift mode. Hence, the rapid expansion of tip leaves fingerprints in the frequency shift, where the instantaneous heating effect cannot be completely compensated by the feedback adjustment of tip height. More accurate positioning can be done by performing nanoscale electric-field imaging based on pulsed-ODMR, as shown in Fig. 2b. In order to induce surface polarization for stabilizing the NV^+, we applied a high positive voltage (typically ≥ +90 V) near the NV for a certain time (typically 10 minutes). Finally, we retracted the tip (or grounded the tip) and checked the fluorescence of NV.

However, it's very time consuming to guide the tip to the atomic color defect with nanoscale precision. In order to control the charge state in a more efficient way, we first scanned the AFM tip within a square frame of 2×2 μm^2 containing the selected NV^- with a high positive voltage (typically ≥+90 V), meanwhile recording the fluorescence signal. A disk-like structure appears in the middle of the scan, indicating the successful charge control between the NV^+ and NV^- (Supplementary Fig. 2c). We then grounded or retracted the tip to turn the NV into dark state (NV^+) (Supplementary Fig. 2d). In order to turn on the same NV, we simply scanned the same region with a negative bias (-90 V). The position of NV could be determined within ~100 nm based on the charge-transition image obtained at small bias voltages (+40 V) (Supplementary Fig. 2e). We found that the polarization/depolarization strength is dependent on the magnitude and the duration time of applied bias voltage.

Supplementary Note 4: Laser power dependence of charge-state transition

During the charge-state control process, the intensity of excitation photons plays

a role. In the charge-state transition curves, the bias threshold reflects the strength of built-in electric field formed by surface electron trapping. Supplementary Fig. 4 shows a group of fluorescence-bias curves on sample A under different laser powers for the same NV center. When the laser power is decreased, the threshold bias for NV^+/NV^0 transition tends to decrease. The reason lies in that the ionization rate of surrounding donor centers is dramatically decreased under small laser power, leading to a small quantity of movable electrons. Thus, the surface trapping of the ionized electrons is less efficient, resulting in a smaller built-in electric field. Meanwhile, we found that the NV^0/NV^- transition becomes much steeper under small laser power, leading to a prominent double-step feature. We note that the fluorescence intensity of the NV^0 is increased after removing the 650-nm LP (inset of Supplementary Fig. 4), due to the large weight of its side band below 650 nm. It is known that the laser excitation can induce interconversion between NV^0 and NV^-, with a larger recombination rate into NV^- under high excitation powers (>50 μW)[5]. In Fig. 3, we used relatively large laser powers to ensure the sufficient signal-to-noise ratio of PL measurements (400~500 μW). Therefore, the NV^0/NV^- transition is likely smeared out, leading to the absence of such a two-step structure.

Supplementary Note 5: Local contact potential difference measurements

In order to confirm the tip-induced surface polarization during the charge-state control, we performed local contact potential difference (LCPD) measurements on the sample B. To ensure good conductivity of the tip, we treated the tip on graphite first by applying bias pulses (+4~10 V in magnitude and ~200 ms in width) or directly poking into graphite surface by several tens of nanometers. After the treatment, we checked the tip conductivity through the tunneling current at low biases. Typically, a tip with good conductivity only needs a small excitation energy for maintaining preset amplitude of tuning fork (~300 pm during tip treatments). Then, we transferred the tip onto the diamond surface and focused on a single NV. The laser was turned off to avoid the change of surface polarization during the LCPD measurements. To obtain LCPD

signals with high signal-to-noise ratio, we need to polarize the selected region under high positive voltages (>+90 V) and large laser powers (>200 μW). The surface polarization before the LCPD measurements in Fig. 4c was induced by a tip bias of +120 V with 1-s integration time per pixel under 420 μW. We polarized a small region of 300×300 nm^2 on the diamond surface and compared the local electrostatic potential inside and outside this region. We didn't perform LCPD measurements on the sample A due to the instability of surface adsorbents on sample A.

Supplementary Note 6: Surface-chemistry dependence of charge-state transition

Since the surface polarization should be very sensitive to the chemical species adsorbed on the diamond surface, we performed a comparison experiment on acid-boiled chips (sample A) and isopropanol-immersed chips (sample B) (see Methods). The AFM images show a much cleaner surface of sample B while the sample A is covered by ~5 nm thick adsorption layers (Supplementary Fig. 6). In this work, we have realized charge-state control both on these two diamond chips (samples A and B) with different surface treatments (Supplementary Fig. 7). For both samples, we found a similar behavior of charge-state transition during the bias ramp (Supplementary Fig. 7, a and c). However, there's a big difference in the transition threshold and the resulted charge states on the two samples. After the surface polarization under the same tip bias (+90 V), we found that the averaged transition voltage of NV^+ to NV^0 on sample A is 27.2±8.6 V, while the one on sample B is 70.3±15.3 V. In addition, the PL spectra show that the NV^- state cannot be reached even under +150 V on sample B, while both NV^0 and NV^- are visible for sample A (Supplementary Fig. 7, b and d). The higher threshold bias indicates a stronger built-in electric field formed by the tip-induced surface polarization and hence more efficient screening of the tip electric field. We recall that the sample B has relatively clean surface without going through acid-boiling or any lithography process. However, for sample A, some adsorbent layers (most likely the water layers) are covered on the diamond surface after the Piranha treatment. Those adsorbents may saturate the surface traps (such as sp^2 defects) through charge transfer

and suppress the surface trapping of photon-ionized electrons from bulk, leading to smaller built-in electric field.

Supplementary Note 7: Estimation of the screening effect in a diamond nanopillar

Since the diamond is a dielectric material with a nonnegligible dielectric constant (~5.7), the electric screening effect on the shallow NVs several nanometers close to the diamond surface should be considered. Here we simulated the electric field imaging by a single shallow NV both in the air, the bulk diamond, and the flat-top nanopillars through COMSOL (Supplementary Fig. 8). We modeled the source of the external electric field using a sharp tip (radius of curvature: 10 nm) with a simple conical shape.

The simulation shows that the field strength of E_z sensed by NV in the diamond bulk is reduced to ~60% of that in the air, and the shielding effect is enhanced when the size of diamond host is decreased. Despite the existence of considerable screening effect, there is no obvious distortion in the electric field imaging within the scanning range of 200×200 nm^2, which is obtained by the NVs hosted in the diamond bulk and the flat-top nanopillar with a diameter of 200 nm (Supplementary Fig. 8a-c and e-g). However, when we used the nanopillar with a 20-nm diameter for the simulation, the distortion effect in the measured electric field imaging is somewhat more prominent (Supplementary Fig. 8d and h). This is because that the diameter of the nanopillar is close to the size of the conical tip apex (10 nm in this simulation). In addition, we found that the screening effect marginally degrades the spatial resolution of electric-field contour imaging (Supplementary Fig. 8e-h). However, the spatial resolution shows weak dependence on the size of the diamond pillar, which maintains the level of <10 nm.

Due to the existence of the charged defects and trapped charges on/near the diamond surface, obtaining the absolute strength of electric field is challenging for the NV in diamond. However, since the NV-based electrometry measures external signals in a quantitative manner, this shortcoming can be possibly circumvented by a pre-calibration procedure. For example, before measuring the target sample by the diamond

tip, a metal tip with well-defined tip apex produced by focused ion beams can be used for obtaining the standard electric-field imaging or providing the point-spread function, which is a general method in other NV-SPM techniques[7,8]. Such a reference image can be compared with the COMSOL simulation by the same tip model. Then, the averaged background shielding effect either from the dielectric diamond host or the surface adsorption layers in ambient conditions can be reasonably estimated through this process. An effective dielectric constant, which may have spatial dependence, can be extracted by the pre-calibration and used for calculating the absolute values of local electric fields.

Therefore, we conclude that the flat-top diamond pillar has no obvious effects on the NV-based electric-field imaging as long as the size of diamond host is far larger than the spatial resolution of NV-based electrometry. Typically, for the diamond tip generally used in scanning magnetometry[6], shallow NVs were grown in a flat-top diamond pillar with a diameter around 200 nm, which corresponds to the optimized size for achieving high-efficiency photon collection and retaining good coherence. We believe that the diamond tip, which has already been extensively used in scanning magnetometry, is also suitable for future applications in scanning electrometry.

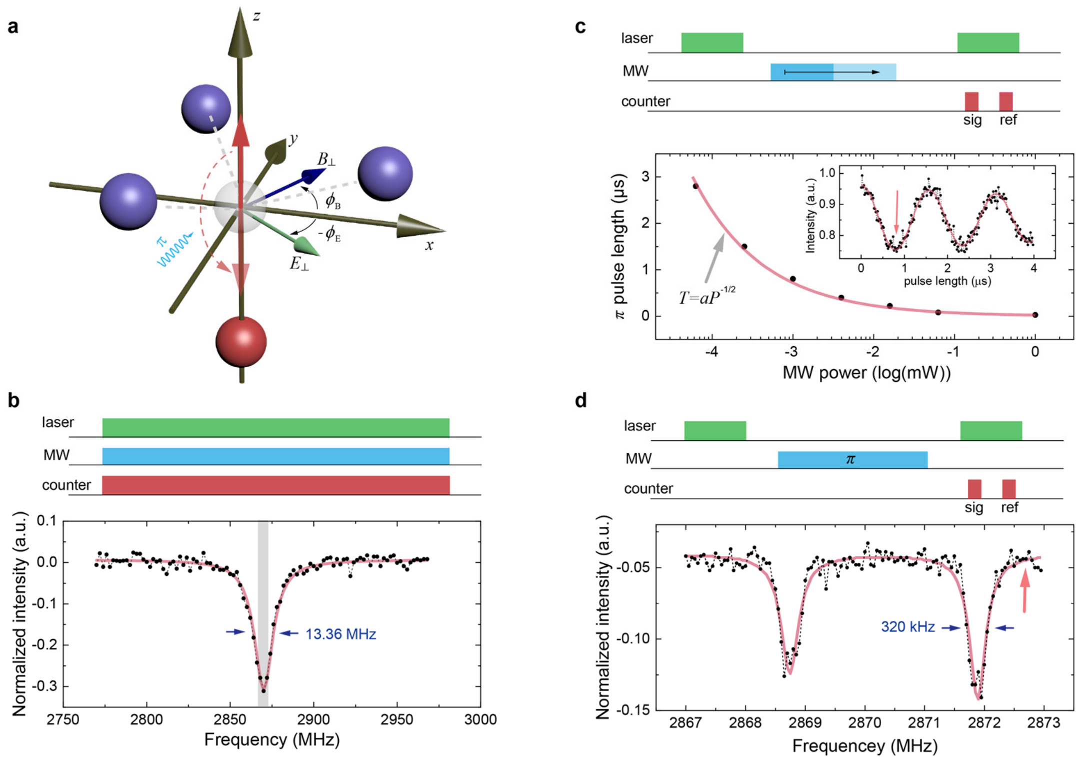

Supplementary Fig. 1 | Pulsed ODMR. a, Schematic graph of NV center, coordinate axes, external fields and π-pulse. The red and transparent balls represent the nitrogen atom and vacancy, respectively, while the violet balls represent the nearest carbon atoms. z axis is along the symmetry axis of NV. **b**, Continuous wave-ODMR under zero fields showing a spectral broadening of 13.36 MHz. The gray region represents the frequency sweeping range of (**d**). **c**, Upper panel: pulse sequence of Rabi oscillation. Lower panel: microwave power dependence of π-pulse length. The data are fitted by $T=aP^{1/2}$, where T is the π-pulse length and P is the laser power. Inset: typical Rabi oscillation with a fitted π-pulse length of 776 ns. The red arrow denotes the position of π-pulse. **d**, A typical pulsed-ODMR showing the hyperfine structure. Upper panel: pulse sequence of pulsed-ODMR. Lower panel: pulsed-ODMR spectra under a small transverse magnetic field (9.6 Gauss). The hyperfine structure is consistent with the N^{15} isotope. The spectral resolution of this NV is 320 kHz. Red arrow denotes the frequency setpoint for scanning field-gradient imaging.

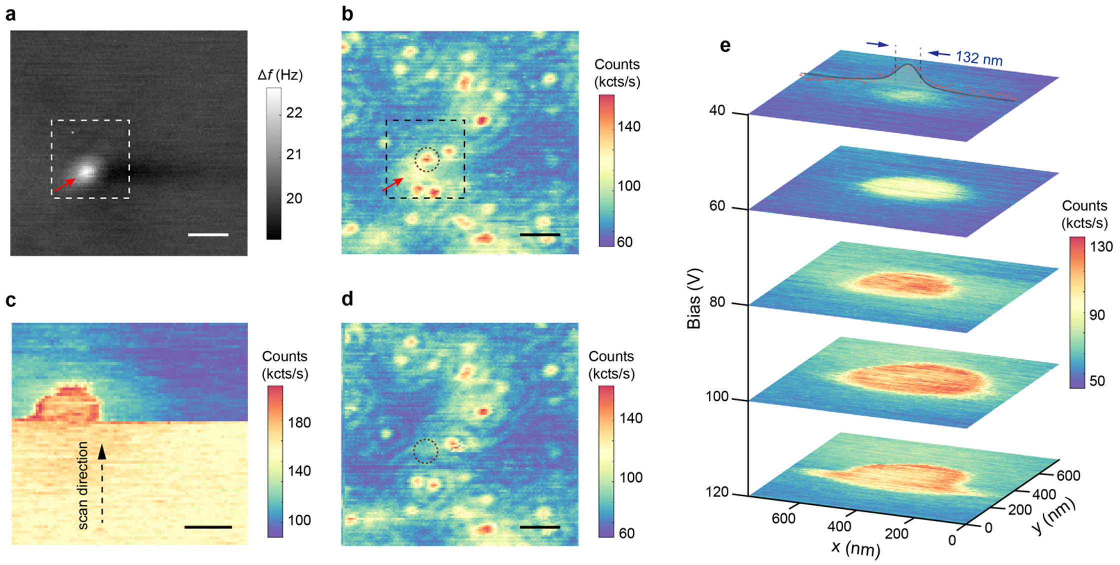

Supplementary Fig. 2 | Process of highly efficient charge-state control. a, Frequency-shift AFM image during scanning the laser spot, showing the tip position (red arrow). **b**, Confocal image simultaneously recorded with (**a**). The tip was guided close to a specific NV center. The red arrow denotes the tip position, which appears as a slightly higher background. The black dashed circle denotes the target NV for charge state control. **c**, Charge-state transition image in the region of (**a**) and (**b**) (denoted by dashed squares). The tip with a high positive bias (+120 V) was scanned around the target NV. A disk-like feature appears in the middle, indicating the successful surface polarization around the NV center and that the charge-state control is enabled. The black dashed arrow denotes the scanning direction. **d**, Confocal image when the tip was retracted, showing the dark state of NV. **e**, Charge-state transition image recorded under different tip biases. The disk-like structure shrinks with decreased bias. The line profile under +40 V was fitted by a Gaussian function, demonstrating a full width at the half maximum (FWHM) of 132 nm. Scale bars in (**a**), (**b**) and (**c**) are 1 μm. Scale bar in (**c**) is 400 nm.

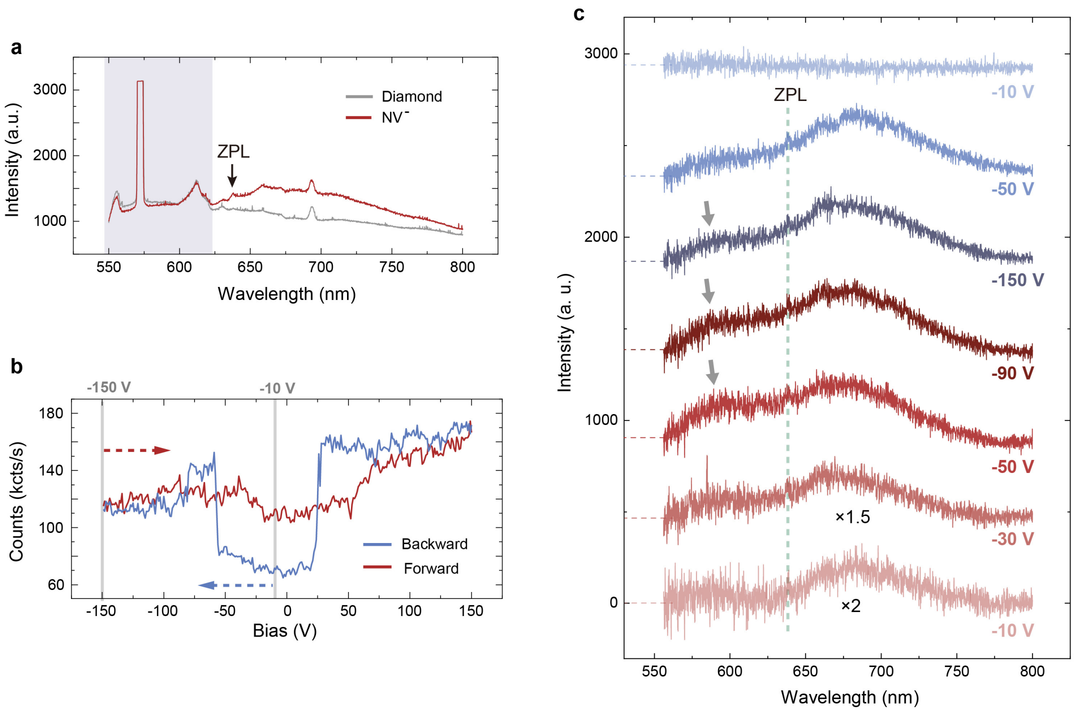

Supplementary Fig. 3 | Supplementary PL spectra. a, PL spectra measured under the 532-nm laser. The background Raman signals (denoted by the grey shadow) from the diamond lattice are very prominent compared to the fluorescence of single NV. There is a severe overlap between the Raman peaks and the ZPL of NV^0, while only the ZPL of NV^- is visible. **b**, Fluorescence-bias curve during a complete control process (same as Fig. 4a). The red and blue dashed arrows denote the direction of bias ramp. **c**, PL spectra (background removed) of a single NV along the negative bias ramp between -10 V and -150 V as shown in (**b**). The series of PL spectra clearly point to the NV^- state, except for the top one (NV^+ state). The green dashed line indicates the ZPL position of NV^- state. No obvious side band of NV^0 state is visible in the series of PL spectra. However, the small shoulder around 600 nm (denoted by grey arrows) might arise from the component of NV^0 state, due to the tip-induced band bending effect under large negative biases. Spectra are offset vertically with respect to the one under -10 V for clarity, where the zero levels are denoted by dashed lines. The PL spectra in (**c**) were recorded under 488-nm excitation laser with 550-nm LP.

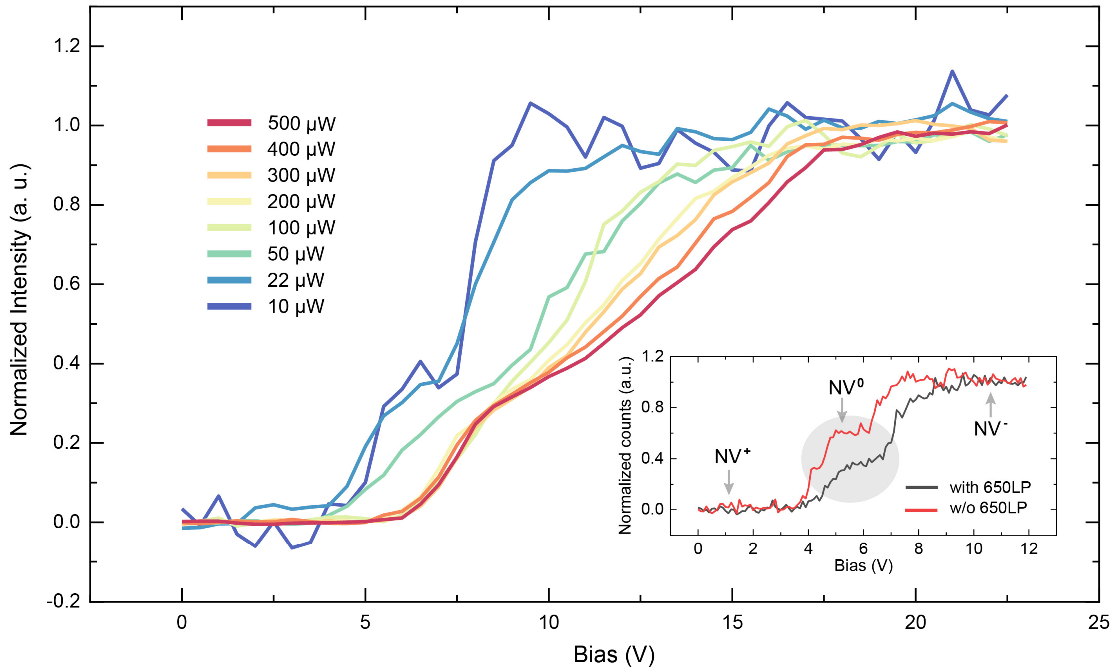

Supplementary Fig. 4 | Laser power dependence on charge state control. Normalized fluorescence-bias curves after averaging out of 4 ramp cycles of a single NV under different laser powers. The data were normalized by the formula: $f_n(V) = [f(V) - f_b]/[f_- - f_b]$, where f_- and f_b represent photon counts of the NV^- and diamond background, respectively. When the laser power is decreased, the NV^+/NV^0 transition threshold tends to decrease. Meanwhile, the NV^0/NV^- transition slope becomes much steeper under small laser powers, leading to a prominent double-step feature. Inset: Normalized fluorescence-bias curves under 10 μW with and without 650-nm LP. The photon counts of NV^0 state is enhanced after removing the LP (highlighted by a grey ellipse), due to the large weight of its side band below 650 nm.

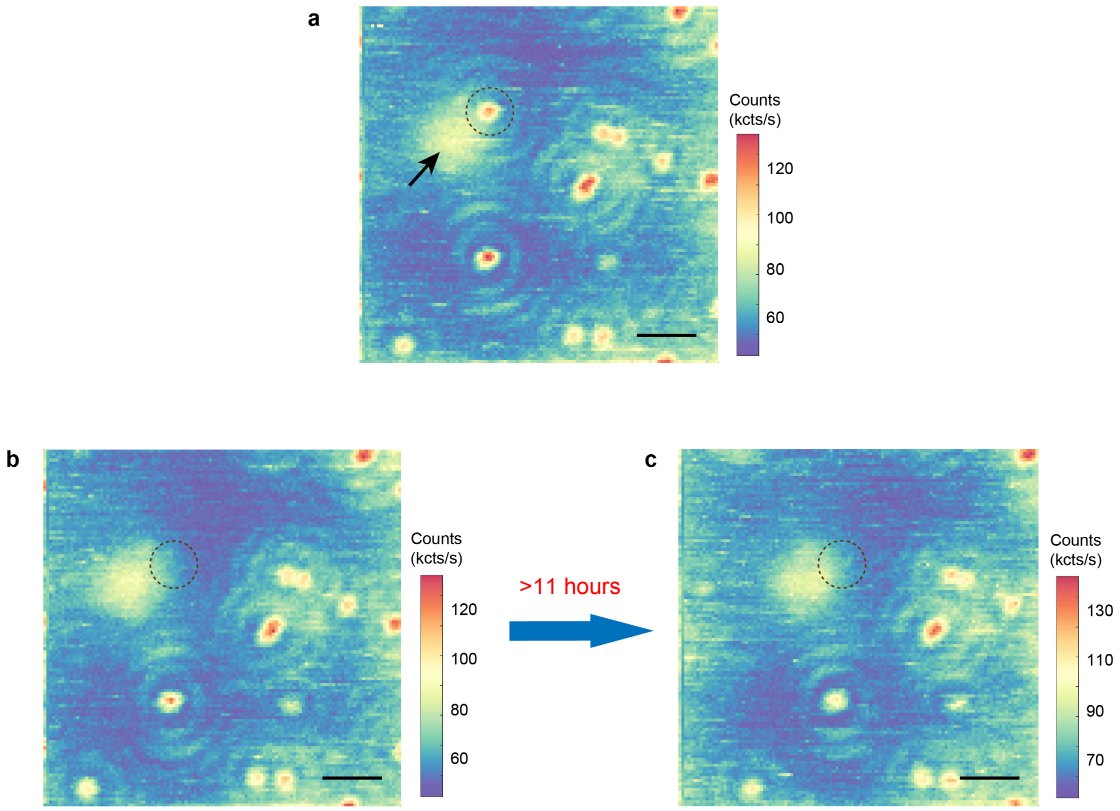

Supplementary Fig. 5 | Long-living NV$^+$ under the thermal equilibrium condition. **a**, Confocal image before charge state control of target single NV$^-$ (highlighted by a black dashed circle). The arrow denotes the position of nearby metal tip, leading to a slightly higher background photon counts. **b**, Confocal image after the charge state control. The bright NV$^-$ was turned into the dark NV$^+$. **c**, Confocal image recorded in the same area as (**b**) after ~11 hours, during which the tip was grounded and the laser was turned off. The NV$^+$ was very stable and did not revive to the bright state (NV$^-$). Scale bar: 1 μm

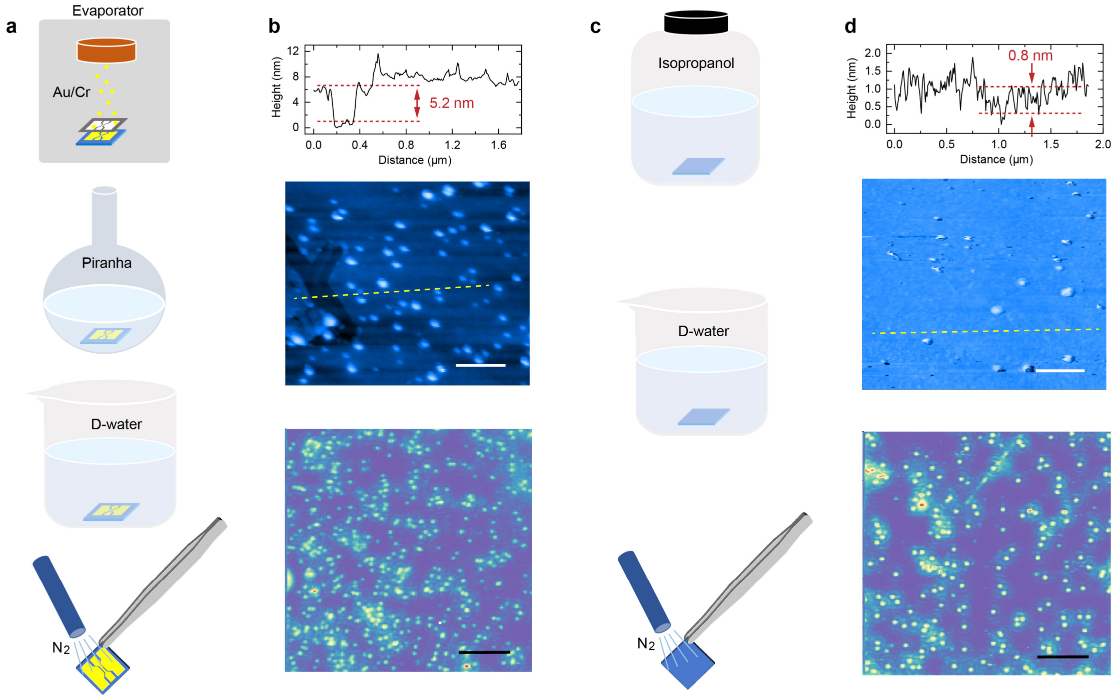

Supplementary Fig. 6 | Preparation and surface characterization of samples A and B. a, Surface preparation of sample A. On-chip waveguide was deposited with a shadow mask. The chip was then acid-boiled, rinsed by D-water (Deionized water) and dried by nitrogen gas. **b**, AFM tomography (middle panel) and typical confocal image (lower panel) within the waveguide gap on the sample A. The line profile (upper panel) shows the surface of sample A is covered by adsorbed layers with a height of ~5 nm. **c**, Surface preparation of sample B. The chip was immersed in isopropanol for several months and then rinsed by D-water and dried by pure nitrogen gas. **d**, AFM tomography (middle panel) and typical confocal image (lower panel) of sample B. The line profile (upper panel) shows a height variation smaller than 1 nm, indicating the relatively clean surface. The uniform distribution of isolated bright spots in the confocal images of both samples suggests the high quality of the implanted NVs. Scale bars in the AFM images: 400 nm. Scale bars in the confocal images: 4 μm.

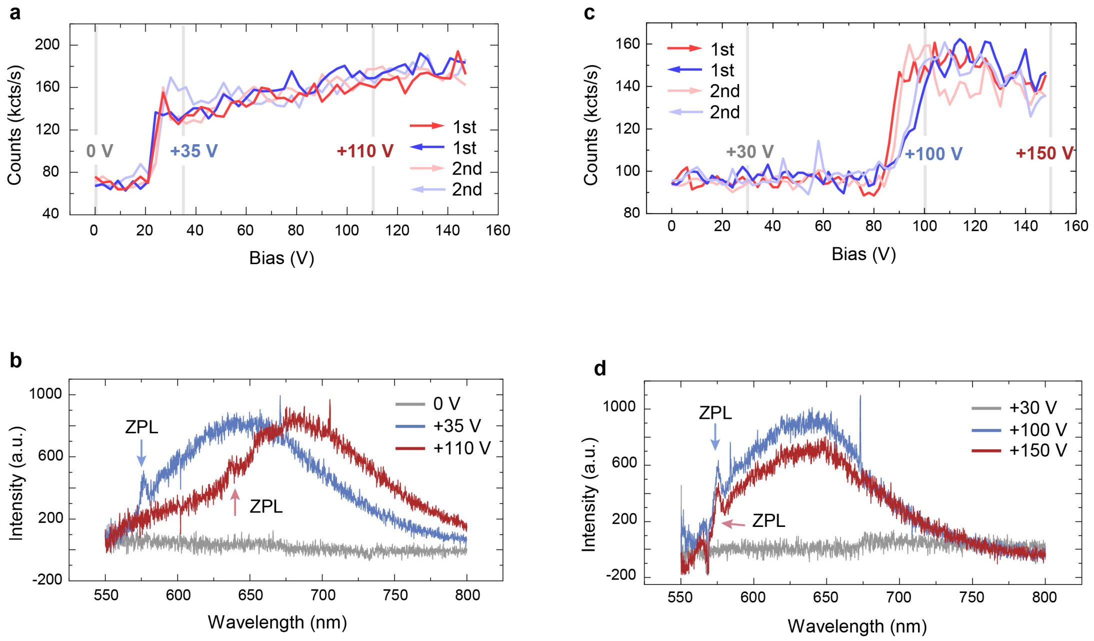

Supplementary Fig. 7 | Surface chemistry dependence on charge state control. a, b, Typical fluorescence-bias curves and corresponding PL spectra of a single NV on sample A. **c, d**, Typical fluorescence-bias curves and corresponding PL spectra of a single NV on sample B. The transition threshold on sample B is much larger than that on sample A (see **a** and **c**). Moreover, the PL spectrum of NV^- state is absent on sample B (**d**), while it is present on sample A (**c**). Such a difference can be attributed to the much stronger built-in electric field near the surface of sample B under the same polarization condition (tip bias: +120 V).

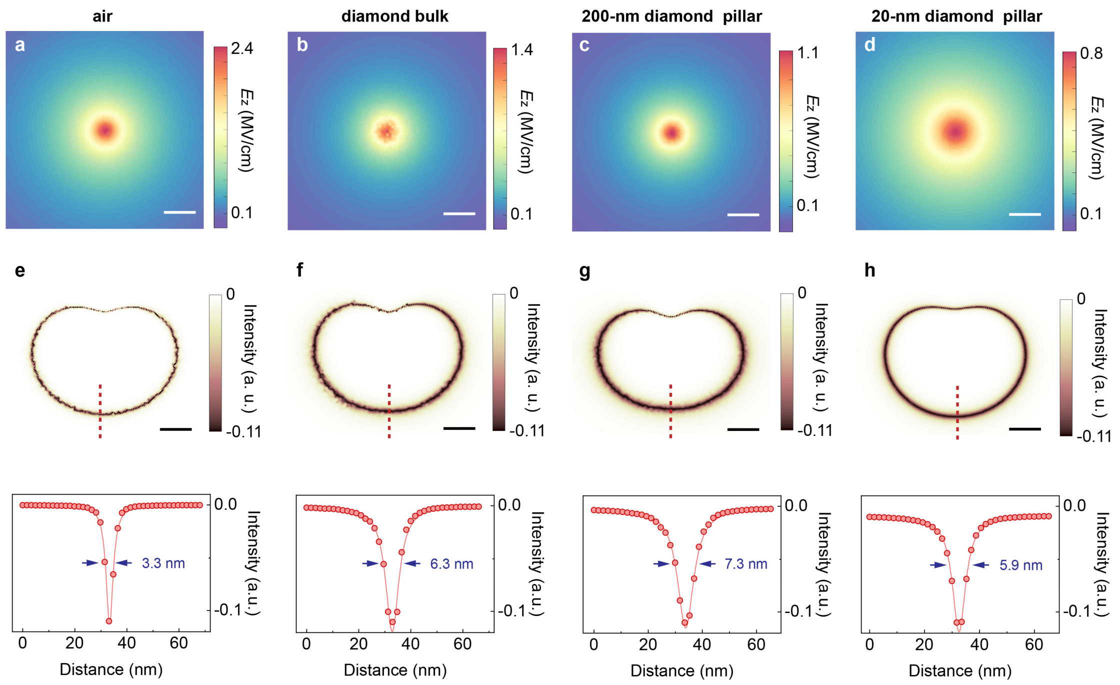

Supplementary Fig. 8 | The screening effect of diamond host on the electric-field imaging. a-d, Simulated E_z distribution of a conical-shape tip sensed by the NV hosted in the air (a), diamond bulk (b), a nanopillar with the diameter of 200 nm (c) and a nanopillar with the diameter of 20 nm (d). The model for simulation is similar as described in Fig. 2a. The NV is in [111] direction of diamond and located at the center of the nanopillar with a depth of 5 nm. We set the tip height as h=5 nm and the tip bias as -16 V. The radius of curvature at the tip apex was set as 10 nm and the divergence angle as ~45°, while the flat-top diamond nanopillar has a height of 400 nm. The dielectric constant of diamond 5.7 was used through all the simulations. The tip was scanned over the diamond surface with a tip height of 5 nm. **e-h,** Simulated pulsed-ODMR mapping obtained from the NV hosted in the air (e), diamond bulk (f), the 200-nm nanopillar (g) and the 20-nm nanopillar (h). The non-spherical shape arises from the fact that the NV axis is not perpendicular to the diamond surface. The line profile in each image shows the measured field gradient by NV, which reflects the spatial resolution of NV-based scanning electrometry. The pulsed-ODMR setpoint was set as 8.5 MHz for (e), 2.9 MHz for (f), 2.8 MHz for (g) and 3.5 MHz for (h), and the full width at half maximum f_{FWHM} of the pulsed-ODMR function was set as 260 kHz. Scale bar: 30 nm.

References

1 Dolde, F. *et al.* Electric-field sensing using single diamond spins. *Nat. Phys.* **7**, 459-463 (2011).

2 Michl, J. *et al.* Robust and Accurate Electric Field Sensing with Solid State Spin Ensembles. *Nano. Lett.* **19**, 4904-4910 (2019).

3 Felton, S. *et al.* Hyperfine interaction in the ground state of the negatively charged nitrogen vacancy center in diamond. *Phys. Rev. B* **79**, 075203 (2009).

4 Oberg, L. M. *et al.* Solution to Electric Field Screening in Diamond Quantum Electrometers. *Phys Rev Appl* **14**, 014085 (2020).

5 Aslam, N., Waldherr, G., Neumann, P., Jelezko, F. & Wrachtrup, J. Photo-induced ionization dynamics of the nitrogen vacancy defect in diamond investigated by single-shot charge state detection. *New J. Phys.* **15**, 013064 (2013).

6 Maletinsky, P. *et al.* A robust scanning diamond sensor for nanoscale imaging with single nitrogen-vacancy centres. *Nat. Nanotechnol.* **7**, 320-324 (2012).

7 Grinolds, M. S. *et al.* Quantum control of proximal spins using nanoscale magnetic resonance imaging. *Nat. Phys.* **7**, 687-692 (2011).

8 Grinolds, M. S. *et al.* Subnanometre resolution in three-dimensional magnetic resonance imaging of individual dark spins. *Nat. Nanotechnol.* **9**, 279-284 (2014).
